# The Influence of the Type of Metal-on-Metal Hip Endoprosthesis on the Clinical, Biochemical, and Oxidative Balance Status—A Comparison of Resurfacing and Metaphyseal Implants

**DOI:** 10.3390/nano15161258

**Published:** 2025-08-15

**Authors:** Aleksander Augustyn, Michał Dobrakowski, Dominika Rokicka, Marta Wróbel, Sławomir Kasperczyk, Krzysztof Strojek, Bogdan Koczy, Tomasz Stołtny

**Affiliations:** 1 District Hospital of Orthopaedics and Trauma Surgery in Piekary Śląskie, Bytomska St. 62, 41-940 Piekary Śląskie, Poland; 2 Department of Biochemistry, Faculty of Medical Sciences in Zabrze, Medical University of Silesia in Katowice, Jordana St. 19, 41-808 Zabrze, Poland; 3 Department of Internal Medicine, Diabetology and Cardiometabolic Disorders, Faculty of Medical Sciences Zabrze, Medical University of Silesia, 41-800 Zabrze, Poland

**Keywords:** metaphyseal hip arthroplasty, hip resurfacing arthroplasty, metal ions, metal-on-metal, oxidative stress

## Abstract

**Introduction:** Despite the increasingly rare use of metal-on-metal articulation, in many countries, there is a large group of patients after hip arthroplasty using this method. The operation of the dysfunctional hip joint using metal-on-metal articulation can be performed by resurfacing or total, stemmed arthroplasty. The aim of this study was to compare the metaphyseal and resurfacing methods in metal-on-metal articulation and its impact on clinical status and variability of oxidative stress parameters, as well as the concentration of chromium and cobalt ions in blood. **Materials and methods:** The first group operated using BHR (Smith & Nephew) and Biomet ReCap-Magnum metal-on-metal hip resurfacing implants. The second group operated using Biomet ReCap-Magnum with a Microplasty metaphyseal stem. Two clinical evaluations were conducted in each patient with the use of SF-12, HHS, and WOMAC-hip examination scale systems. The concentrations of metal ions in blood and their impacts on the antioxidant system were also determined twice using various oxidative stress markers. **Results:** The study included 61 males. The mean values of the Cr (*p* = 0.019) and Co (*p* = 0.009) ion concentrations were higher in patients after resurfacing arthroplasty. A higher intensity of oxidative stress (activities of sulphydryl groups, malondialdehyde, and lipofuscin) was observed in the resurfacing group compared with the metaphyseal group. **Conclusions:** The resurfacing hip implant in the metal-on-metal articulation, to a greater extent than the metaphyseal one, elevates the concentration of Cr and Co ions in the blood and is associated with oxidative stress and the functioning of the antioxidant system.

## 1. Introduction

Metal-on-metal arthroplasty of the hip returned to wide use in the second half of the 1990s. Systems using it were dedicated especially to young, active patients as a potentially effective alternative to implants with friction surfaces including polyethylene [1].

The initial, extremely optimistic clinical results associated with their use [2,3,4] were quickly confronted with emerging negative reports on local and systemic adverse reactions associated with the presence of elevated concentrations of metal ions [5,6].

In view of the above, and despite the fact that individual implants were withdrawn from the market due to allegations of defectiveness described in the literature, there is still a large group of patients in many countries who live with good functional outcomes, despite the higher metal ion release compared with other types of implants [7,8].

Dysfunctional hip joint surgery using metal-on-metal articulation can be performed using a resurfacing technique or total stemmed arthroplasty.

According to a recently conducted analysis of the types of primary hip arthroplasties performed and the methods of their fixation by Klug et al., it is possible to determine the trend of development of hip joint arthroplasty. Based on the obtained data, it was found that the frequency of classic cementless and metaphyseal arthroplasty is increasing, while the frequency of resurfacing and cemented arthroplasty is decreasing [9].

The elevated concentration of released metal ions at the local and systemic level is a source of limited biocompatibility of both mentioned variants of endoprostheses functioning in this particular type of articulation [10].

The distribution of metal ions and particles in the body is associated with systemic adverse effects, including teratogenicity, organ toxicity, carcinogenesis, and immunotoxicity [11].

The aim of this study was to compare the metaphyseal and resurfacing methods in metal-on-metal articulation and its impact on clinical status and variability of oxidative stress parameters, together with the concentrations of chromium (Cr) and cobalt (Co) ions in blood.

## 2. Materials and Methods

### 2.1. Description of the Studied Groups and Implants Used

**Figure 1:**
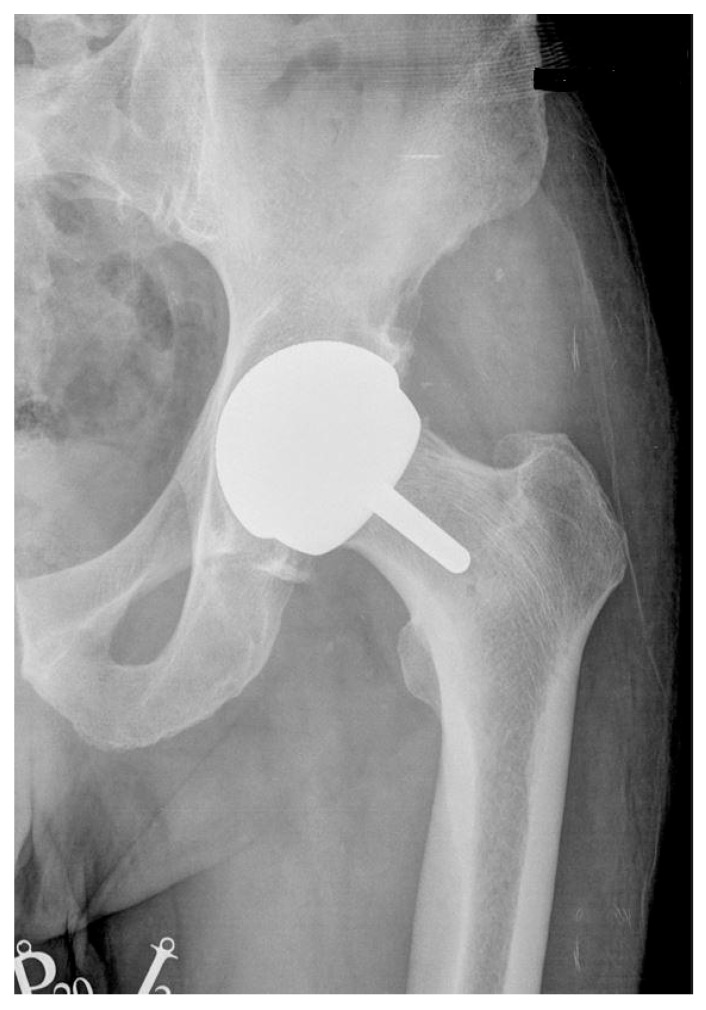
X-ray scan showing the hip operated with use of BHR (Smith & Nephew, Watford, UK).

**Figure 2:**
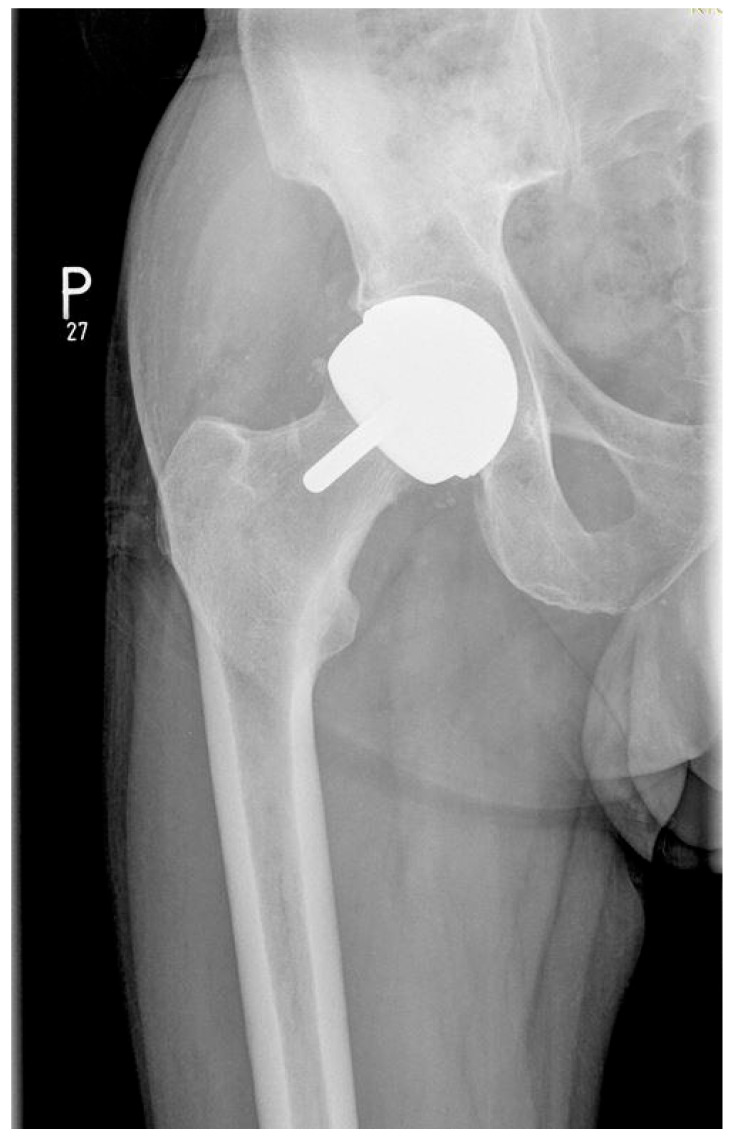
X-ray scan showing the hip operated with use of ReCap-Magnum (Biomet, Warsaw, IN, USA).

In group I, consisting of 26 males, hip resurfacing arthroplasty was conducted in metal-on-metal bearing using BHR (Smith & Nephew, Watford, UK; Figure 1), n = 21, and ReCap−Magnum (Biomet, Warsaw, IN, USA; Figure 2), n = 5 systems.

The BHR system consists of a porous hydroxyapatite coated acetabular component enabling cementless implantation and a cemented femoral component. Both components consist of a cast high-carbon cobalt–chromium alloy with a 2 mm diameter increment for the acetabular component and a 4 mm diameter increment for the femoral component [12].

The metallurgy of the Biomet ReCap-Magnum resurfacing system is a high-carbon (>0.2%) cobalt–chromium cast without heat treatment. The surface roughness is less than 0.5 μm. The ReCap femoral component is a hemisphere on top of a cylindrical stem with a grit-blasted cobalt–chromium lower surface. The implants are slightly thinner than most others on the market [3].

**Figure 3:**
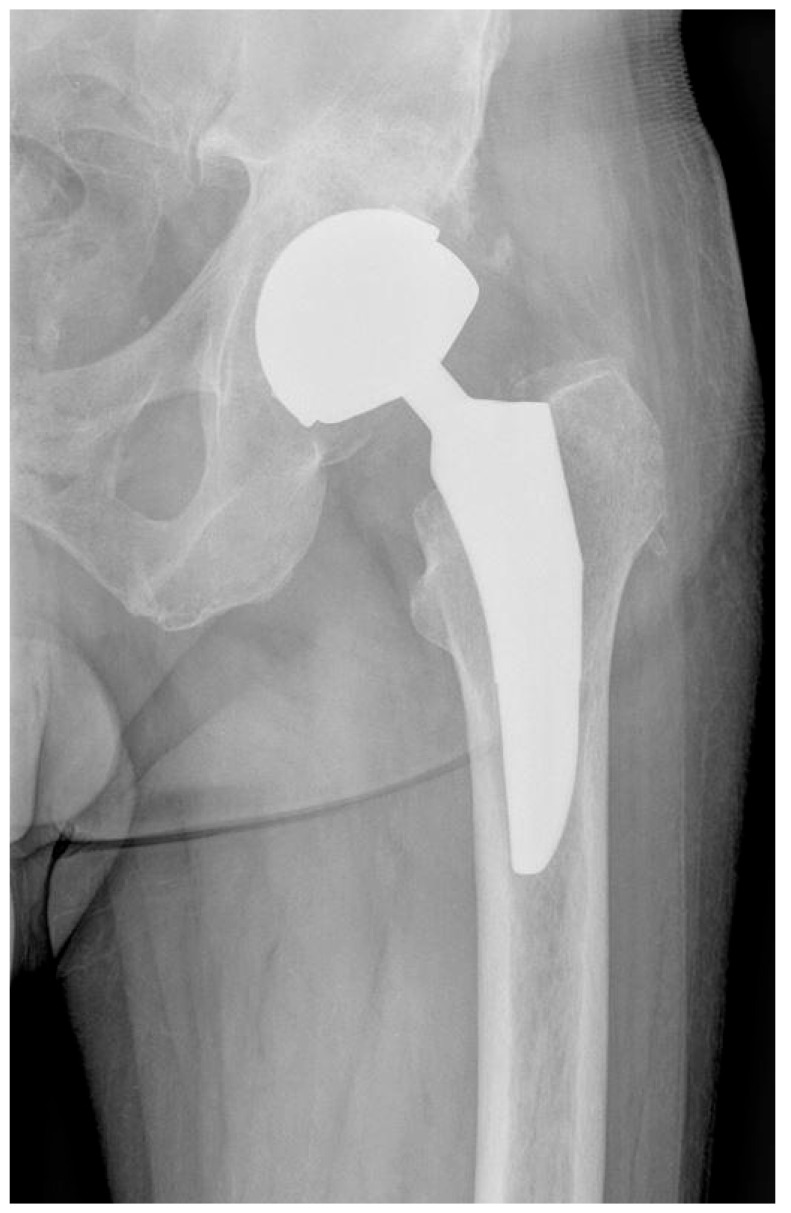
X-ray scan showing the hip operated with use of ReCap-Magnum with a Microplasty metaphyseal stem (Biomet, Warsaw, IN, USA).

In group II, consisting of 35 males, metaphyseal arthroplasty of the hip joint was performed in metal-on-metal bearing using the ReCap-Magnum systems with a Microplasty metaphyseal stem (Biomet, Warsaw, IN, USA; Figure 3), n = 35.

The TaperLoc^®^ Microplasty™ femoral component is a titanium collarless stem with a flat, tapered-wedge geometry demonstrating a proximal, titanium, porous plasma-sprayed surface. The collarless feature allows for optimal component seating and enhanced rotational stability. A lateralized neck offset option is available along with seven neck length options [13].

### 2.2. Research Methodology

Patients from each study group appeared for two follow-up visits after the arthroplasty surgery, during which they were examined in detail clinically and biochemically from a blood sample.

#### 2.2.1. Clinical Evaluation

SF-12, HHS, and WOMAC-HIP scales along with physical examinations were used for the clinical assessment of patients. The SF-12 scale for evaluating the quality of life in the physical and mental category is a recognized and widely acclaimed tool [14]. The HHS scale assesses joint mobility, deformities, functioning, and pain, with a maximum of 100 points [15]. WOMAC-HIP stands as a proven, multilayered tool for evaluating impairment for patients with arthrosis of the hip joint—the worst result being 96 points and the best being 0 points [16].

The clinical examination included the measurement of the length of the lower limbs (with possibility of lengthening or shortening the operated limb). Quadriceps femoris muscle strength was evaluated with the usage of a proven 5-point Lovette scale. A goniometer was used for the assessment of the range of motion: adduction, abduction, flexion, and external and internal rotation movements in the operated hip joint. Pain was determined using the VAS scale, which uses a straight line, with one end representing “no pain” and the other “worst pain imaginable.” Patients mark a point on the line corresponding to their pain level, and the distance from the “no pain” end is measured in centimeters. This measurement is then interpreted as a numerical indicator of pain intensity [17]. Another tool used to assess pain was a five-point pain scale, in which the patient reports the intensity of pain in the operated hip from 0 to 5.

#### 2.2.2. Biochemical Evaluation

Biochemical tests were performed at District Hospital of Orthopaedics and Trauma Surgery in Piekary Śląskie, at Medical University of Silesia in Katowice—Facility of General Biochemistry, and at the Laboratory of the Lead and Zinc Smelting Facility in Miasteczko Śląskie.

The concentrations of chromium (Cr) and cobalt (Co) were determined using a Thermo Fisher Scientific, Oxford, UK, ICE 3400 Atomic Absorption Spectrometer, with GFS35Z graphite furnace, with an automatic sample feeder, deuterium correction on a QuadLine lamp, and correction of the background based on the transverse Zeeman effect. The cobalt concentration was evaluated according to Schwingel Ribeiro at a wavelength of 242.5 nm, and chromium was evaluated according to Huang at a wavelength of 357.9 nm. Ion concentrations are given in μg/L [18,19].

The concentration of ceruloplasmin (CER) in serum was evaluated using the reaction with p-phenylenediamine as described by Richterich [20]. The sulfhydryl group (SH) concentration was evaluated according to Koster et al. [21] and presented in μmol/L.

Total antioxidant capacity (TAC) in serum was evaluated according to Erel [22] and given in mmol/L, and total oxidant status (TOS) in serum was evaluated as outlined by Erel [23] and given as μmol/L. Lipid hydroperoxides (LPH) were determined in serum in accordance with Arab et al. [24], using xylene orange and given in μmol/L.

Fluorometrical evaluation in erythrocytes as described by Ohkawa et al. was used to determine the level of malondialdehyde (MDA), which is a by-product of lipid peroxidation [25]. The concentration was given in µmol/g Hb. Lipofuscin (LPS) levels were evaluated using Tsuchida et al.’s approach [26]. Its levels are given as relative units (RU) in erythrocytes per gram of hemoglobin (Hb) and as RU/L in serum (100 R is equal to the fluorescence of a 0.1 mg/mL solution of quinidine sulfate in sulfuric acid).

Superoxide dismutase (SOD) activity was determined in erythrocytes and serum in accordance with Oyanagui [27]. SOD enzymatic activity was expressed in nitric units. When nitric ion production is inhibited by SOD by 50%, it means the activity of SOD equals 1 nitric unit. SOD activity was normalized to milligrams of Hb (NU/mg Hb). The activity of catalase (CAT) in erythrocytes was evaluated according to the kinetic method of Johansson et al. [28] and shown as kU/g Hb.

Glutathione reductase (GR) activity in erythrocytes was evaluated as described by Richterich [20] and given in μmoles of NADPH utilized per g Hb per minute (IU/g Hb).

Glutathione S-transferase activity (GST) in erythrocytes was determined in accordance to the kinetic method of Habig et al. [29] and given as μmoles of thioether produced per g Hb per minute (mIU/g Hb). The activity of Glutathione peroxidase (GPX) in erythrocytes was determined according to Paglia and Valentine [30] and given as micromoles of NADPH oxidized per g Hb per minute (IU/g Hb).

### 2.3. Statistical Analysis

Statistical analysis was performed using Statistica 10.0 PL Software and MS Excel 2019. For continuous variables the mean and standard deviation (SD) were determined. To verify normality, Shapiro–Wilk’s test was used. To verify the homogeneity of variances, Levene’s test was utilized. A *t* test, a *t* test with a separate variance, or a Mann–Whitney U test were used for the statistical comparisons between the groups. Changes were considered statistically significant at the significance level of *p* ≤ 0.05. The Spearman rank correlation coefficient was used to measure the correlation.

## 3. Results

The results for 61 patients (n = 26 + n = 35) are shown in Table 1, Table 2, Table 3, Table 4, Table 5 and Table 6. The first follow-up visit occurred at an average of 64.1 months after the procedure in group I and 61.6 months in group II. The second follow-up visit occurred approximately 6 months after the first. The results are given as an average of both visits.

Clinical features of both groups are displayed in Table 1.

**Table 1:** General features of the study groups with implant measurements (data presented as mean from both follow-up visits ± SD).

Parameter	Group I		Group II		Change %	*p*-Value
	n = 26		n = 35			
	Mean	SD	Mean	SD		
Body weight (kg)	93.2	18.8	91.6	16.9	−2%	0.743
Height (m)	1.78	0.07	1.75	0.06	−2%	0.060
BMI (kg/m^2^)	29.3	5.01	30.0	4.85	2%	0.640
Time after surgery (months)	64.1	6.4	61.6	5.88	−4%	0.112
Age at the time of surgery (years)	54.2	9.1	59.7	9.5	10%	**0.027**
Age at the time of control visit (years)	59.6	9.21	64.7	9.46	8%	**0.042**
Head size diameter (mm)	52.9	1.98	50.2	3.04	−5%	**<0.001**
Cup size diameter (mm)	58.9	1.98	56.1	3.09	−5%	**<0.001**

In bold—*p* ≤ 0.05.

The advantage of this study are largely homogenous groups in terms of basic parameters such as weight or height.

Clinical indications for using a particular prosthesis type are behind the age difference.

Significantly larger head and cup sizes for resurfacing implants have been noted (*p* =< 0.001).

The range of motion values of the hip joint after surgery along with the strength of the muscle are displayed in Table 2. The HHS score is comparable in both groups. In group I a significative inclination toward shortening of the operated limb in comparison with the other side (*p* = 0.004) was noted.

**Table 2:** Evaluation of the clinical status of the operated hip, muscle strength, and range of motion (data presented as mean from both follow-up visits ± SD).

Parameter	Group I		Group II		Change %	*p*-Value
	n = 26		n = 35			
	Mean	SD	Mean	SD		
Flexion	108.5	14.0	106.0	12.2	−2%	0.467
Abduction	38.8	5.0	36.1	6.4	−7%	0.079
External rotation	27.5	7.2	29.7	8.2	8%	0.279
Internal rotation	16.2	9.4	13.6	8.0	−16%	0.252
Adduction	31.2	7.8	29.9	6.2	−4%	0.473
HHS sum	85.6	15.1	81.3	19.4	−5%	0.355
Symmetry/asymmetry	−0.42	0.77	0.56	1.53	-	**0.004**
Lovett muscle strength	4.81	0.49	4.89	0.32	2%	0.458

In bold—*p* ≤ 0.05.

As presented in Table 3, no advantages for any of the groups concerning joint mobility or patient functionality were found. WOMAC-HIP and SF-12 clinical evaluation scales were applied to assess the influence of the used hip implant on particular aspects of the patient’s life. In our work, no propensity for higher pain in either group after hip arthroplasty was observed. The scores for each WOMAC-HIP subscale are summed up, with a possible score range of 0–8 for stiffness, 0–20 for pain, and 0–68 for daily activity. Higher scores on the WOMAC-HIP correspond with worse stiffness, pain, and functional limitations. The SF-12 uses a norm-based scoring system, meaning scores are interpreted relative to a reference population. A score of 50 indicates the average health status in the overall population. Scores that exceed 50 represent better-than-average health-related quality of life. Scores under 50 indicate below-average health status.

**Table 3:** Pain, mood, and clinical evaluation of the studied groups (data given as mean from both follow-up visits ± SD).

Parameter	Group I		Group II		Change %	*p*-Value
	n = 26		n = 35			
	Mean	SD	Mean	SD		
WOMAC stiffness	0.83	1.20	1.13	1.78	36%	0.457
WOMAC pain	2.71	3.52	3.07	4.43	31%	0.483
WOMAC daily activity	11.2	15.1	11.3	15.8	14%	0.711
WOMAC-sum	14.9	19.3	14.5	21.7	19%	0.632
SF12-physical health	15.8	3.31	15.1	3.70	−4%	0.497
SF12-mental health	22.9	3.48	21.1	4.56	−8%	0.102
VAS 1–10	1.40	1.40	1.67	1.90	19%	0.547
Six-point pain scale	0.65	0.80	0.91	0.95	40%	0.262

As demonstrated in Table 4, the Cr ion concentration was elevated in each group. An elevated level of Co ions in group I was observed. Higher concentrations of both Co (*p* = 0.009) and Cr (*p* = 0.019) ions in group I is a noteworthy aspect. A concentration of Cr ions below 1.4 μg/L is considered normal, whereas for Co ions, normal was considered below 1.8 μg/L.

**Table 4:** Concentration levels of metal ions in studied groups (data presented as mean from both follow-up visits ± SD).

Parameter	Group I		Group II			*p*-Value
	n = 26		n = 35			
	Mean	SD	Mean	SD		
Cr ions	4.09	7.66	2.04	1.31		**0.019**
Co ions	2.69	3.69	1.58	0.93		**0.009**

In bold—*p* ≤ 0.05.

As presented in Table 5, in group I compared with group II, the following parameters were noted: elevated intensiveness of oxidative stress expressed as a higher concentration of MDA in erythrocytes (*p* = 0.014), a lower concentration of SH (*p* = 0.033) in serum, and elevated concentrations of LPS in the serum (*p* = 0.001) and in erythrocytes (*p* ≤ 0.001). GPx activity was 23% higher, and catalase activity was 25% lower; SOD activity on subsequent visits remained close to stable.

**Table 5:** Oxidative stress and antioxidants enzymes in the studied population (data displayed as mean from both follow-up visits ± SD).

Parameter	Group I		Group II		Change %	*p*-Value
	n = 26		n = 35			
	Mean	SD	Mean	SD		
CER (mg/dL)—serum	35.3	8.09	34.8	6.81	−1%	0.788
SH (umol/L)—serum	209	41.0	243	76.2	16%	**0.033**
TAC (mmol/L)—serum	0.99	0.10	1.06	0.16	6%	0.085
TOS (umol/L)—serum	8.62	2.92	7.55	3.01	−12%	0.170
LPH (umol/L)—serum	4.55	1.60	4.04	2.07	−11%	0.301
MDA (umol/g)—erythrocytes	0.40	0.05	0.36	0.06	−9%	**0.014**
LPS (RF)—serum	810	221	586	257	−28%	**0.001**
LPS (RF/g)—erythrocytes	1565	573	725	192	−54%	**<0.001**
SOD (NU/mL)—serum	18.2	1.58	18.3	1.48	1%	0.796
SOD (NU/mg)—erythrocytes	165	21.6	162	17.87	−2%	0.528
MnSOD (NU/mL)—serum	10.8	1.93	10.1	0.99	−6%	0.077
CuZnSOD (NU/mL)—serum	7.40	1.24	8.18	1.40	11%	**0.027**
CAT (IU/g)—erythrocytes	547	98.7	413	57.5	−25%	**<0.001**
GR (IU/g)—erythrocytes	7.66	1.42	8.09	1.74	6%	0.311
GST (IU/g)—erythrocytes	0.19	0.05	0.17	0.04	−11%	0.118
GPX (IU/g)—erythrocytes	54.4	9.69	67.0	4.75	23%	**0.000**

In bold—*p* ≤ 0.05. CER—ceruloplasmin; SH—sulfhydryl groups; TAC—total antioxidant capacity; TOS—total oxidant status; LPH—lipid hydroperoxides; MDA—malondialdehyde; LPS—lipofuscin; SOD—superoxide dismutase; CAT—catalase; GR—glutathione reductase; GST—glutathione S-transferase; GPX—glutathione peroxidase.

Table 6 presents a positive correlation of the higher concentration of Cr ions and the elevated SH concentration in serum. A positive correlation of higher Co ions and increased CAT and LPS in erythrocytes was noted. Additionally, a negative correlation between elevated Co ions and GPX concentration in erythrocytes was observed.

**Table 6:** Correlations of studied metal ions and oxidative stress (Spearman R, *p* < 0.05).

Parameter	Cr (μg/L)	Co (μg/L)
SH (μmol/g)—serum	0.26	NS
CAT (IU/g)—erythrocytes	NS	0.32
LPS (RF)—erythrocytes	NS	0.35
GPX (IU/g)—erythrocytes	NS	−0.35

SH—sulfhydryl groups; CAT—catalase; LPS—lipofuscin; GPX—glutathione peroxidase; NS—not significative; Cr—chromium; Co—cobalt.

## 4. Discussion

Despite the increasing frequency and availability of surgical treatment of symptomatic hip osteoarthritis, in 2060 the demand for the number of hip joint arthroplasty procedures is predicted to increase by as much as 37.7% compared with 2018 [31].

The choice between metaphyseal or resurfacing hip arthroplasty in younger patients with symptomatic hip osteoarthritis is a matter of ongoing debate and depends on many factors.

The National Joint Registry for England and Wales reported that in 2010, resurfacing procedures accounted for 14% of all hip arthroplasties in patients under the age of 55 years, with significantly poorer clinical outcomes and a higher rate of revision required, particularly in females [32].

Reported potential advantages of resurfacing arthroplasty include more physiological biomechanical conditions of the hip joint, lower dislocation rates, maintenance of normal bone mineral density of the proximal femur, increased range of motion and activity level after surgery, greater improvement in general health status, and reduced mortality. On the other hand, disadvantages include a longer learning curve, longer surgical time, limitations related to certain anatomical variants, adverse reactions to elevated metal ion concentrations, and a narrower range of proper placement of endoprosthesis components [33].

In our study, we obtained data on satisfactory results of patients operated on using the resurfacing method, including reduction in pain and improvement in clinical tests, but the range of motion was not significatively different from group II, despite the use of larger head and cup sizes.

A significant problem revealed by our study is the difficulty in correction of the preoperative limb asymmetry with a tendency for the operated limb to remain shortened in the case of the use of a resurfacing endoprosthesis. There are two factors behind this phenomenon. First, the implantation sequence begins with the femoral component, the so-called “head-cap”, and subsequent adjustment of the acetabulum size to it. This order forces the surgeon to apply larger diameter acetabular miller cutters than the size of the bone socket would suggest, which may cause excessive immersion of the acetabular component along with a shortened hip offset, ultimately shortening the limb [34]. The second factor is the design of the resurfacing endoprosthesis, which does not have a separate head element placed on a Morse taper, unlike the stemmed endoprosthesis, and whose appropriate adjustment provides an additional possibility of compensating the postoperative limb length.

The above observations seem to be backed by the study by Parry et al. and the results obtained therein, comparable to ours, in a group of 86 patients after arthroplasty using the resurfacing method compared with the stemmed endoprosthesis [35].

In their study, Ford et al. presented clinical outcomes and risk of revision in a group of 314 patients after BHR (Smith & Nephew) hip arthroplasty over a 5- and 10-year follow-up period. The implant survival rates of 97.2% and 93.8% were associated with satisfactory clinical outcomes and a greater likelihood of returning to a high level of physical activity compared with stemmed hip arthroplasty [36]. We obtained similar results in our study in the resurfacing implant group.

Satisfactory results of the Biomet ReCap-Magnum hip resurfacing arthroplasty were obtained by Kiran et al. in a study of 72 operated hips in a long-term follow-up period of at least 10 years. The implant survival rate was 97% along with a significative improvement in the clinical status of patients in recognized assessment scales (OHS, UCLA) [37].

Cr and Co ions release as an outcome of friction between the metallic surfaces of endoprostheses or as a result of corrosion, which brings the release of metal hydroxides, phosphates, and oxides [38].

The reason for the higher concentrations of Cr and Co ions in patients in group I may be the fact that the used acetabular and femoral implants are statistically significantly larger in size than the metaphyseal implants. As a consequence, the articulating surface has a proportionally larger area within which ions are released due to friction [39]. However, there are discrepancies among authors regarding the size of the implant diameter in metal-on-metal articulations as the main factor of increased concentration of Cr and Co ions. In our study, we did not obtain direct correlations between higher concentrations of the tested ions and implant size. The structure of the implant, the spatial relations of the femoral and acetabular components after placement in the bone, and the metallurgy used in production also seem to play an important role.

The imbalance between the generation of reactive oxygen forms and their elimination by the antioxidant system is the basis of the condition known as the oxidative stress. A lot of researchers claim that this phenomenon is what causes the toxic effects of Cr and Co on the organism [40,41].

Being redox-active metals, cobalt and chromium are a source of reactive oxygen forms, e.g., catalyzing the Fenton reaction [42]. This results in stimulation of the oxidative stress, which, among others, may lead to damaging of cell membranes in the mechanism of lipid peroxidation [43].

The markers of increased oxidative stress and the associated aging process include LPS [44] and MDA [45]. In our study, the concentrations of LPS in serum and erythrocytes and MDA in erythrocytes were significantly elevated in the resurfacing group compared with the metaphyseal group, in which lower concentrations of Cr and Co ions and a higher mean age were noted.

Elevated antioxidant concentrations or the increased activity of antioxidant enzymes amid stimulated oxidative stress should be interpreted as an adaptation of the organism, in accordance with the hormesis theory [46]. Therefore, it is appropriate to adopt that the activity of antioxidant enzymes may be dependent on various factors, which are the result of opposing mechanisms at the time of evaluation. First, their activity may be lower as a result of the direct toxic effect of a given element or compound, while in contrast it may be higher as an outcome of the activation of protective mechanisms in response to amplified oxidative stress. This is the manner the SH activities observed in our study should be interpreted. They were significantly lower in group I but showed a positive correlation with increased concentration of Cr ions. Sulfhydryl groups (SH, thiols) are effective antioxidants that can maintain the correct structure of proteins and protect cells and tissues against damage caused by oxidative stress [47].

When it comes to catalase, its activity is higher in group I compared with group II, and it also correlates positively with elevated Co ions. The higher activity of this enzyme in the group that also has higher concentrations of Cr, Co, and LPS can be explained by the above-mentioned adaptive defense mechanism of the organism [44].

Other studies have shown that Cr and Co can directly bind to proteins, including antioxidant enzymes, and compete with other metals for binding sites in active sites, which may lead to their impaired activity [48,49]. These reports explain the reduced activity of GPx observed in group I compared with group II. Furthermore, confirming the above thesis, in our study we observed a weak but still negative correlation between GPx activity and Co concentration.

In both study groups, it was possible to achieve a reduction in pain, an increase in joint mobility, an improvement in patient’s essential functions regarding movement, and an improvement of quality of life—factors that are the main determinants of successful hip arthroplasty. To analyze the effect of the type of hip replacing implant on the above-mentioned aspects of the patient’s life, we used the SF-12, HHS, and WOMAC-HIP clinical assessment scales. Neither group achieved an advantage in terms of joint mobility, deformation, and patient functioning, although it is worth noting that the patients in group II were statistically significantly older. Comparable clinical results of both types of implants studied may also indicate optimal qualification of patients for the use of the appropriate implant.

The strength of our study was the considerable homogeneity of the studied groups, taking into account weight, height, and BMI, as well as comparable time of the follow-up visits after surgery.

The interpretation of the obtained values of oxidative stress parameters and antioxidant system function is difficult due to the fact that the average age in the metaphyseal group was higher in comparison with the resurfacing group. This along with the difference in quantities in both groups should be seen as a limitation of this study.

In order to obtain as homogeneous groups as possible, we decided to limit the inclusion to male patients in the study, which also may be seen as a limitation.

## 5. Conclusions

Implantation of a resurfacing endoprosthesis in the setting of osteoarthritis of the hip joint is associated with a limited possibility of compensating for the initial shortening of the operated limb.

Patients operated on using the resurfacing method in metal-on-metal bearing demonstrate higher concentrations of cobalt and chromium ions in the blood compared with the metaphyseal method, which, however, does not translate into worse postoperative clinical results.

The resurfacing implant in the metal-on-metal bearing is associated with higher induction of oxidative stress than the metaphyseal one and modifies the activity of the antioxidant system, which is primarily due to the increased concentration of Cr and Co ions.

## Data Availability

The authors confirm that the data supporting the findings of this study are available within the article. Raw data concerning the patients are being held in the District Hospital of Orthopaedics and Trauma Surgery in Piekary Śląskie, Bytomska St. 62, 41-940 Piekary Śląskie, Poland.
